# GDF15 as a key disease target and biomarker: linking chronic lung diseases and ageing

**DOI:** 10.1007/s11010-023-04743-x

**Published:** 2023-04-24

**Authors:** Yang Wan, Jianhua Fu

**Affiliations:** Department of Pediatrics, Shengjing Hospital of China Medical University, Shenyang, China

**Keywords:** GDF15, Chronic lung disease, Mitochondrial dysfunction, Senescence, SASP

## Abstract

Growth differentiation factor 15 (GDF15), a member of the transforming growth factor-beta superfamily, is expressed in several human organs. In particular, it is highly expressed in the placenta, prostate, and liver. The expression of GDF15 increases under cellular stress and pathological conditions. Although numerous transcription factors directly up-regulate the expression of GDF15, the receptors and downstream mediators of GDF15 signal transduction in most tissues have not yet been determined. Glial cell-derived neurotrophic factor family receptor α-like protein was recently identified as a specific receptor that plays a mediating role in anorexia. However, the specific receptors of GDF15 in other tissues and organs remain unclear. As a marker of cell stress, GDF15 appears to exert different effects under different pathological conditions. Cell senescence may be an important pathogenetic process and could be used to assess the progression of various lung diseases, including COVID-19. As a key member of the senescence-associated secretory phenotype protein repertoire, GDF15 seems to be associated with mitochondrial dysfunction, although the specific molecular mechanism linking GDF15 expression with ageing remains to be elucidated. Here, we focus on research progress linking GDF15 expression with the pathogenesis of various chronic lung diseases, including neonatal bronchopulmonary dysplasia, idiopathic pulmonary fibrosis, chronic obstructive pulmonary disease, and pulmonary hypertension, suggesting that GDF15 may be a key biomarker for diagnosis and prognosis. Thus, in this review, we aimed to provide new insights into the molecular biological mechanism and emerging clinical data associated with GDF15 in lung-related diseases, while highlighting promising research and clinical prospects.

## Introduction

Growth differentiation factor 15 (GDF15), initially termed macrophage inhibitory factor-1 in 1997, is a stress response cytokine that belongs to the transforming growth factor (TGF)-β superfamily. It is also known as non-steroidal anti-inflammatory drug induced gene (NAG-1), placenta transforming growth factor-β, prostate-derived factor, and placental bone morphogenetic protein [1–3]. GDF15 serves as a general biomarker for several diseases, with its serum level being used to predict all-cause mortality in conditions such as heart failure and cancer. GDF15 has also been reported to be a senescence-associated secretory phenotype (SASP) protein, indicating a role as an autonomic regulator of cellular senescence.

Chronic lung diseases, including bronchopulmonary dysplasia, idiopathic lung fibrosis, chronic obstructive pulmonary disease, and pulmonary hypertension, may be associated with an accelerated ageing of the lungs. Mounting evidence suggests that GDF15, senescence, and the pathogenesis of chronic lung diseases may be interlinked. Herein, we summarise the status of current research on the role and underlying mechanism(s) of GDF15 in chronic lung diseases.

### Synthesis, secretion, and distribution of GDF15

**Fig. 1:**
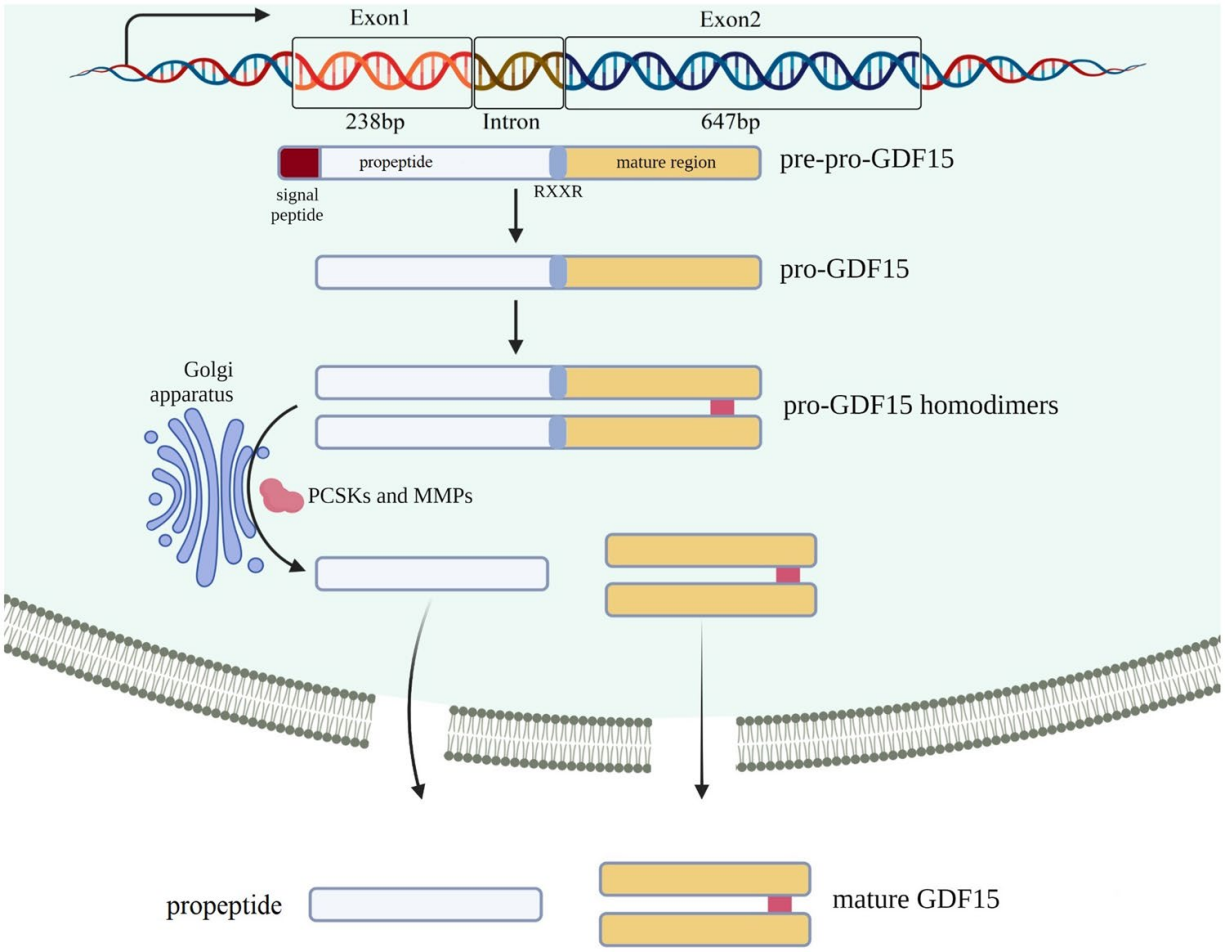
Human *GDF15* includes 2 exons and 1 intron. Inactive human GDF15 pre-pro-protein in the cytoplasm is dimerised by a specific disulphide bond, cleaved at the RXXR cleavage site in the Golgi apparatus, and secreted as mature GDF15. *PCSK*: Proprotein convertase subtilisin/kexin; *MMP*: Matrix metalloproteinase

Human *GDF15*, comprising 2 exons and 1 intron, is situated on chromosome 19p13.1–13.2 and comprises a total sequence length of 2746 base pairs [4, 5], as shown in Fig. 1. The GDF15 protein is approximately 35 kDa in size and includes a cysteine knot in the C-terminal domain formed by eight intrachain disulphide bonds, which is the hallmark of the TGF-β superfamily. However, mature GDF15 is distinguished by a unique disulphide bonding configuration in its cysteine knot core [6] and is thus considered as a divergent member of the TGF-β superfamily. Human GDF15 pre-pro-protein contains 308 amino acid residues [7], comprising a 29-amino acid signal peptide, a 167-amino acid pro-peptide at the N-terminus, and a 112-amino acid mature region at the C-terminus [8]. The GDF15 precursor protein undergoes disulphide‐linked dimerisation through a cysteine residue and is then cleaved at the RXXR cleavage site by proprotein convertase subtilisin/kexins and matrix metalloproteinases in the Golgi apparatus [7, 9].

In healthy individuals, GDF15 is expressed most abundantly in the placenta, followed by the prostate, kidney, colon, liver, and lung; it may also be expressed in the brain, heart, pancreas, gastrointestinal tract, and bone marrow at lower levels. In physiological states, GDF15 is only weakly expressed with a median circulatory level of 762 ng/L (interquartile range, 600–959) in healthy elderly individuals (median age of 65 years) [10]. However, GDF15 is highly expressed in the serum of pregnant women, with its concentration gradually increasing during pregnancy. GDF15 in the placenta and amniotic fluid may promote placental formation and help maintain pregnancy through its immunosuppressive effect [11]. The expression of GDF15 in other tissues may also increase under certain pathological states such as inflammation [12], tumorigenesis [7], oxidative stress [13], ischaemia, anoxia, and hypoxia [14], as well as during ageing [15].

### Expression and regulation of GDF15

**Fig. 2:**
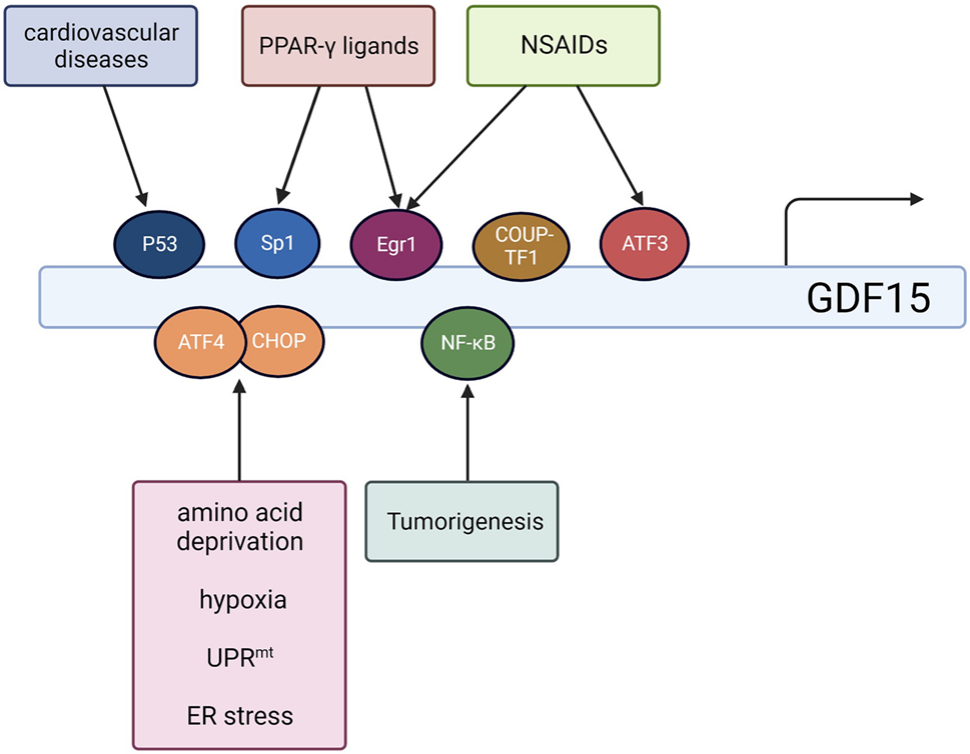
Overview of regulation of GDF15 expression. *PPAR-γ*: Peroxisome proliferator-activated receptor γ; *NSAID*: Non-steroidal anti-inflammatory drugs; *P53*: Tumour protein 53; *Sp1*: Specificity protein 1; *COUP-TF1*: COUP transcription factor 1; *ATF*: Activating transcription factor; *CHOP*: C/EBP homologous protein; *NF-κB*: Nuclear factor-κB; *UPR*^*mt*^: Mitochondrial unfolded protein response; *ER*: Endoplasmic reticulum

GDF15 expression is up-regulated by various transcription factors, as shown in Fig. 2. The region upstream of the GDF15 promoter consists of several basic transcription factor-binding sites, including specificity protein 1, early growth response protein 1 (Egr-1), p53, and COUP transcription factor 1 [4]. In the promoter of GDF15, Egr-1 and specificity protein 1 bind to the same DNA sequence [16]. Known stress signals, such as amino acid deprivation, hypoxia, mitochondrial dysfunction, and endoplasmic reticulum stress, play a role in activating transcription factor 4 (ATF4) to form a heterodimer with C/EBP homologous protein (CHOP) via the phosphorylation of elF2α, resulting in the regulation of GDF15 transcription [17]. However, hypoxia- and anoxia-induced GDF15 expression during tumour growth depends on the level of promoter histone methylation rather than p53/hypoxia-inducible factor 1 (HIF-1) expression [15, 18]. Hypoxic exposure may drive the transcription of *GDF15* by activated pancreatic endoplasmic reticulum kinase–eukaryotic initiation factor 2 alpha signalling pathway; this triggers the up-regulation of CHOP, which binds to the GDF15 promotor directly [14]. Furthermore, the level of GDF15 has been shown to increase following treatment with non-steroidal anti-inflammatory drugs; hence, GDF15 has also been referred to as NAG-1, which is induced by Egr-1 and ATF-3 rather than cyclooxygenase/p53 [19–21]. In addition, peroxisome proliferator-activated receptor γ ligands act as positive regulators of GDF15 via interactions with Egr-1 and ATF-3/4 [22–24]. In cardiovascular diseases, GDF15 expression is stimulated by C-reactive protein through the p53 pathway in endothelial cells [25]. In addition, nuclear factor (NF)-κB can directly regulate GDF15 to evade macrophage surveillance during the early stages of tumour development [26].

However, the specific receptors and downstream mediators of the GDF15 signalling pathway in various tissues have not been identified to date. As a member of the TGF-β superfamily, GDF15 was initially considered to interact with a highly conserved receptor superfamily comprising type I and type II receptors. For example, GDF15 exerts its effects on food consumption and energy metabolism by interacting with the receptor TGF-β receptor II in the hypothalamus [27]. However, it has been demonstrated that glial cell-derived neurotrophic factor family receptor α-like protein (GFRAL), which is expressed only in the brain stem, is the only known orphan receptor that shows a high degree of affinity to GDF15 [6, 28–30]. As a transmembrane cell surface protein, GFRAL must interact with its RET receptor on the cell surface to initiate GDF15-specific signal transduction [6, 31]. The complex formed by the binding of GDF15 to GFRAL induces autophosphorylation of the intracellular domain of RET and activates signalling pathways, such as ERK1/2, Akt, FOS, and PLC-γ [32], while not affecting the Smad pathway [4]. GDF15 has been proposed to contribute to anorexia, cachexia, and body weight control via its interactions with these receptors. Moreover, Suriben et al. [33] recently demonstrated that suppression of GFRAL signalling with the therapeutic antagonistic monoclonal antibody 3P10 may reverse GDF15-induced excessive lipid oxidation and prevent cancer-related cachexia. This suggested that GDF15 may elicit lipolysis via the peripheral sympathetic axis, leading to reduced adipose, body, and tissue weights as well as muscle function [33].

The receptors for GDF15 in other tissues and organs remain unidentified. Although TGF-β receptors I and II are reportedly expressed in the lungs, it is unclear whether GDF15 interacts with them the same way as that observed in dendritic cells [34, 35]. GDF15 may be involved in promoting the senescence of respiratory epithelial cells induced by cigarette smoke exposure through the ALK1/Smad1 pathway [36]. GDF15 shows an anti-cardiac hypertrophy effect via the Smad2/3 pathway [37], whereas in cardiomyocytes cultured with GDF15, it exerted a pro-hypertrophic effect via the Smad1 pathway [38]. In cervical cancer, GDF15 binds to the ErbB2 receptor and promotes the proliferation of tumour cells by up-regulating cyclin D1 and cyclin E1 expression and down-regulating p21 expression through the PI3K/Akt and MAPK/ERK signalling pathways [39]. Therefore, future exploration and elucidation of receptor and signalling pathways in tissues other than the brain tissues, under different pathological conditions, might be important.

### GDF15 and stress response

GDF15 acts as a stress-induced cytokine during tissue injury, hypoxia, and stimulation by pro-inflammatory cytokines as well as other stimuli or stressors to maintain cellular and tissue homeostasis. The most well-characterised stimuli include oxidised low-density lipoprotein, growth factors, interleukin (IL)-1β, tumour necrosis factor (TNF)-α, angiotensin II, macrophage colony-stimulating factor, and TGF-β [4, 40]. Hsiao et al. [41] reported that the expression of GDF15 in the liver apparently and swiftly increased in an animal model of partial hepatectomy and carbon tetrachloride-induced liver injury. Zimmers et al. [42] found up-regulated GDF15 expression in mouse models of kidney and pulmonary injury, suggesting that GDF15 induction is a broad cell injury response. GDF15 expression also increases rapidly with cardiovascular injuries, such as myocardial ischaemia/reperfusion [43], dilated cardiomyopathy [37], and heart failure. Xu et al. [44] reported that GDF15, as a newly identified sympathetic regulator, protects against myocardial hypertrophy by inhibiting norepinephrine-induced epidermal growth factor receptor transactivation.

Therefore, GDF15 appears to exert different effects under different conditions. For example, GDF15 exerts anti-inflammatory effects by evading macrophage activation and NF-κB activity, although the mechanism is still not fully understood [45]. Transgenic mice overexpressing GDF15, which were injected with a lipopolysaccharide (LPS), showed lower mortality than wild-type mice, whereas *Gdf15*-knockout mice presented a higher mortality than wild-type mice [46]. Furthermore, GDF15 treatment reduced the mortality rate in the model of inflammation induced by LPS, poly(I:C), or D-galactosamine [12, 47]. Moreover, several lines of evidence suggest that GDF15 may play a protective role under septic conditions. Luan et al. [12] found that GDF15 induced by acute inflammatory injury drives the metabolism of hepatic triglycerides by directing sympathetic outflow to the liver, which was presumed to be mediated by GFRAL-expressing neurons. Conversely, the knockout of *Gdf15* protected mice from caecal ligation and puncture-induced abdominal sepsis [48]. GDF15 also blocks various cytokines, including interferon-γ, IL-6, monocyte chemoattractant protein-1, and TNF-α [4, 43]. In human nasal epithelial cells, GDF15 is regulated by ATF-4 and inhibits the LPS-induced secretion of inflammatory cytokines and mucin 5AC through the PI3K/Akt pathway [24].

### GDF15 and ageing

Senescence is a cellular stress response to molecular damage, characterised by irreversible cell cycle prolongation. Senescent cell arrest results in the formation of a complex secretome, known as the senescence-associated secretory phenotype (SASP). In recent years, a link between GDF15 and senescence has become more evident. A Swedish cohort study, involving a group of 876 male patients aged 35–80 years and a group of 324 twins aged 63–93 years, found that the serum GDF15 level may serve as an independent indicator of all-cause mortality [49]. The authors of that study showed that the serum GDF15 level, similar to telomere length, could predict lifespan independent of the genetic background of an individual. Pence et al. conducted a study which investigated the relationship between circulating GDF15 levels in older adults and indices of age-related monocyte dysfunction, suggesting a potential causal link between GDF15 and age-related decline in immune function [50].

GDF15, the core SASP protein known in humans, is one of the most highly secreted proteins by fibroblasts or epithelial cells among secretory SASPs [51]; however, the specific molecular mechanism underlying its involvement in ageing remains unclear. By activating the ALK1/Smad1 pathway, GDF15 promotes cellular senescence induced by radiation via the reactive oxygen species-mediated p16 pathway in human endothelial cells [52] and facilitates the senescence of airway epithelial cells as induced by cigarette smoke exposure [36]. Another study showed that female transgenic mice overexpressing GDF15 lived longer than wild-type mice [53].

Furthermore, GDF15 is associated with mitochondrial dysfunction [54, 55]. The mitochondria play a key role in the process of ageing, and mitochondrial dysfunction is one of the distinguishing features of senescence. Unfolded or misfolded proteins may accumulate in mitochondrial compartments under cellular stress, resulting in the up-regulation of mitochondrial chaperone protein expression as encoded by nuclear genes. The mitochondrial unfolded protein response (UPR^mt^) is a retrograde transcriptional response that helps misfolded proteins return to normal conformation and ensures that newly synthesized proteins fold correctly. Therefore, the UPR^mt^ is a compensatory mechanism that helps identify, combat, and recover from mitochondrial dysfunction, and it maintains mitochondrial homeostasis together with other mitochondrial stress response pathways, such as mitophagy or mitochondrial dynamics [56, 57]. During mitochondrial stress, UPR^mt^ not only regulates the transcription of mitochondrial genes, such as *ATF4, ATF5*, and *CHOP* [56, 58–60], but also influences the production of stress-responsive molecules known as mitokines, which include GDF15, fibroblast growth factor 21 (FGF21), and mitochondrial-derived peptides [61]. In a *Crif1*(mitoribosomes)-knockout mouse model, aberrant mitochondrial oxidative phosphorylation induces the CHOP-dependent transcription of *Gdf15* after UPR^mt^ activation [62, 63]. However, recent studies have also demonstrated that activation of 5´AMP-activated protein kinase, a key protein for regulating mitochondrial function, can lead to an increase in circulating and hepatic GDF15 levels, independently of CHOP [64]. It has also been suggested that GDF15 may play a protective role by restoring metabolic homeostasis [65, 66]. GDF15 alleviates steatosis of hepatocytes by inhibiting mitochondrial damage and reducing the release of dsDNA from mitochondria to cytosol [67]. In SH-SY5Y cells exposed to rotenone, up-regulated GDF15 affects PGC1α by regulating p53 and then reduce mitochondrial damage and apoptosis, and this process depends on the phosphorylation of Akt/mTOR [68]. GDF15 may also protect mitochondrial function by regulating mitochondrial membrane potential and oxygen consumption of immortalized mouse hippocampal neuronal cells through the PI3K-Akt signal pathway [66]. In addition, the level of GDF15 decreased in the subcutaneous adipose tissue and in vitro-differentiated adipocytes of elderly women, which was negatively correlated with the mRNA expression level of lipogenic genes and was related to mitochondrial dysfunction [68]. Therefore, it can be inferred that GDF15 may be an indicator of mitochondrial dysfunction associated with senescence and age-related diseases.

### Role of GDF15 in chronic lung diseases

#### Bronchopulmonary dysplasia

Bronchopulmonary dysplasia (BPD) is the most common form of chronic lung disease (CLD) in premature infants of gestational age < 28 weeks or birth weight < 1200 g, especially those who require oxygen inhalation or mechanical ventilation during treatment. Since BPD was first described in 1967, advances in integrated management techniques for this condition have effectively improved the survival rate of premature infants. However, its incidence has not declined, and BPD survivors are at risk of a variety of chronic sequelae, including persistent respiratory symptoms, pulmonary function injury, neurodevelopmental disorders, pulmonary hypertension, and post-neonatal death [69].

The primary pathological features of BPD are a reduction in alveolar number, increase in alveolar volume, simplification and irregularity of the alveolar structure, narrowing of the alveolar septum, and abnormal morphology of the pulmonary micro-vessels, which may in turn lead to an abnormal alveolar structure. It is speculated that these changes may be due to arrested development of lung tissue during the vesicular to alveolar phase, thus highlighting the characteristics of a new type of BPD, namely pulmonary stagnation and pulmonary microvascular dysplasia [70]. Current research on the pathogenesis of BPD mainly focuses on the damage and abnormal repair of the pulmonary epithelial barrier following lung injury, DNA damage, and the role of epigenetics in the pathogenesis of BPD, mainly involving mechanisms such as apoptosis and autophagy.

Early damage to the neonatal lung, such as that caused by BPD, can affect different ageing pathways, such as DNA damage, telomere attrition, epigenetic alterations, proteostatic imbalance, mitochondrial dysfunction, cellular senescence, and altered intercellular communication, thereby resulting in premature lung ageing in adults and the early onset of chronic lung disease later in life [71]. Various ageing-related molecular pathways are also associated with neonatal BPD, including TGF-β1-induced connective tissue growth factor expression, the ataxia telangiectasia‐mutated/p53‐dependent pathway, the insulin-like growth factor 1/Akt/mTOR signalling axis, and hyperoxia-induced DNA methylation and histone acetylation changes [71–77]. Hyperoxia can induce the senescence of lung cells, including lung epithelial cells [75, 78], smooth muscle cells of the airways [79, 80], and fibroblasts [81]. As previously described, GDF15 participates in the cellular stress response pathway, which can be induced by hyperoxia exposure, whereas ageing is a protective response to stress, leaving cells in a non-proliferative state that triggers the development of a harmful pro-inflammatory SASP [71]. Hyperoxia has been shown to considerably induce GDF15 expression in the lung [82], especially in epithelial and endothelial cells [83] (Table 1). In addition, the increase in GDF15 expression under hyperoxic conditions may be a response to oxidative stress, and GDF15 knockout could also decrease cell survival and increase reactive oxygen species production [83, 84]. The level of GDF15 in the umbilical cord blood of full-term neonates (3095 ± 191 pg/mL) accounts for 25% of maternal blood levels in the third trimester of pregnancy; however, it is several times higher than that in adults [85], and it is now evident that neonatal GDF15 is derived from the new-born rather than the placenta [85, 86]. Almudares et al. showed that the level of GDF15 negatively correlated with gestational age (i.e., decreased with age) and that the level of GDF15 is directly or indirectly associated with adverse respiratory outcomes in premature infants [87]. Therefore, exploring the role of GDF15 in the pathogenesis of the alveolarisation and lung development dysfunction in BPD has high research value.

**Table 1:** Functional role of GDF15 in response to diverse lung diseases

Disease	Cell type	Functional role	References
Bronchopulmonary dysplasia	Epithelial and endothelial cells	Responds to oxidative stress	[82–84]
Idiopathic lung fibrosis	Epithelial cells	Exerts protective effect in lung fibroblasts/promotes epithelial cell ageing/telomere dysfunction/promote ferroptosis	[35, 87, 95]
Chronic obstructive pulmonary disease	Epithelial cells	Induces cellular senescence/promotes lung inflammation after cigarette smoke exposure/activates EMT after cigarette smoke exposure	[36, 110, 111]
Pulmonary hypertension	Vascular endothelial cells	Induces angiogenesis/prevents endothelial cell apoptosis/causes muscle atrophy	[112, 114, 115, 116]
COVID-19	Endothelial cells	Causes iron metabolism disorder/endothelial inflammation	[127, 129, 133]

#### Idiopathic lung fibrosis

Idiopathic pulmonary fibrosis (IPF) remains an irreversible and progressive fatal disease. It is characterised by the accumulation of extracellular matrix proteins and fibroblast proliferation, leading to chronic pulmonary remodelling and respiratory failure [88]. Studies have revealed that IPF is an ageing-related disease in which the senescence of lung cells plays a major role in the pathogenesis, and the senescence of alveolar epithelial cells promotes fibrosis by generating an SASP [89]. The expression of GDF15 in the human IPF lung is increased, along with increased levels in the bronchoalveolar lavage fluid and plasma, with pulmonary epithelial cells proven to be the main source of GDF15 in this condition [35, 88]. A study involving 108 patients with IPF and 31 healthy controls in China [90] found that the serum level of GDF15 in patients with acute exacerbation of IPF was elevated and that the protein and mRNA levels of GDF15 in IPF lung tissues were significantly increased. Additionally, immunohistochemical staining showed that GDF15 expression in the cytoplasm of type II alveolar epithelial cells was moderately positive. Radwanska et al. co-stained GDF15 with AT II cells marker ProSurfactant protein C (PSPC) in human IPF and healthy lungs [91], and their results also confirmed that GDF15 is expressed in alveolar epithelial type II (ATII) cells. In addition to being involved in promoting epithelial cell ageing, GDF15 exerts a protective effect on lung fibroblasts. Zhang et al. [35] found a connection between up-regulated GDF15 expression in alveolar epithelial type 2 cells and telomere dysfunction in IPF. Thus, GDF15 may play different roles in different lung cell types.

Lambrecht et al. [92] reported that GDF15 can also serve as a marker for the degree of lung damage in systemic sclerosis and that it is associated with the occurrence of fibrosis via the activation of fibroblasts and M2 macrophages. Moreover, the level of GDF15 in vivo is associated with the number of diseased organs in addition to the lung and is thus linked to the severity of the disease. GDF15 participates in immune recruitment in the lungs, activates fibroblasts, and ultimately leads to fibrosis via its direct involvement in the expression of pro-inflammatory cytokines and chemokines (such as IL-6 and CCL2). Additionally, low expression of caveolin-1 in the IPF lung weakens the inhibitory effect of the TGF-β receptor, thereby activating the TGF-β signalling pathway, leading to the excessive production of extracellular matrix and eventually the occurrence of pulmonary fibrosis [93–95]. GDF15 may aggravate the inflammatory response of the lung tissue and accelerate the process of pulmonary fibrosis by promoting ferroptosis, which may be related to the ability of members of the TGF-β superfamily to promote ferroptosis in tumour cells [96]. However, GDF15 has also been proposed as a potential therapeutic agent for IPF, as it could ameliorate pulmonary fibrosis by inhibiting the TGF-β signalling pathway [97]. Therefore, although the mechanism of GDF15 in pulmonary fibrosis is not yet clear, current evidence and technology enable its application as a biomarker for the diagnosis and prognosis of IPF and show its potential as a therapeutic target for IPF.

#### Chronic obstructive pulmonary disease

Chronic obstructive pulmonary disease (COPD) accounts for more than 3 million reported deaths globally each year, and the associated morbidity and mortality are expected to rise in the future. Persistent respiratory symptoms and progressive airflow obstruction are the hallmarks of COPD [98]. As a sensitive marker of cardiopulmonary stress, GDF15 has no known specific diagnostic function in different diseases such as heart failure, pneumonia, COPD, nephropathy, and septicaemia [99], although its expression is markedly increased in patients with acute exacerbations of COPD [100, 101]. Compared with healthy subjects and patients with asthma, patients with COPD show a high GDF15 level in the serum, which is negatively correlated with exercise levels [102]. In a 9-year study of 413 patients with COPD, a high level of plasma GDF15 was independently associated with higher exacerbation rates, higher mortality, and a more significant decrease in the forced expiratory volume in 1 s and forced vital capacities [103]. Therefore, the GDF15 level may be correlated with the severity, deterioration, and prognosis of COPD. In a cohort study of 694 smokers without clinical cardiovascular disease, the level of GDF15 in the plasma independently contributed to the risk of subclinical coronary atherosclerosis [104].

Smoking and occupational exposure to smoke are the leading causes of COPD [98]. Cigarette smoke exposure increases GDF15 expression in airway epithelial cells and induces cellular senescence by activating the ALK1/Smad1 pathway, with significant increases in early senescence marker p21, late senescence marker p16, and HMGB1 levels [36]. This is consistent with the observation of increased cellular senescence in Clara cells, alveolar type II cells, endothelial cells, and leukocytes from smokers or cigarette smoke-exposed mice [72, 105–108], suggesting that the accumulation of senescent cells in the lungs may play a key role in the pathogenesis of COPD. GDF15 was found to regulate MUC5AC expression in respiratory epithelial cells exposed to cigarette smoke by activating the PI3K/Akt signalling pathway [109]. Another study using a mouse model of cigarette smoke exposure showed that the knockout of *Gdf15* could reduce pulmonary inflammation, and that an increase in T and B lymphocytes in the airway and lung tissue was considerably attenuated after 4 weeks of cigarette smoke exposure [110]. The human rhinovirus (HRV) is the most common virus causing acute exacerbations of COPD. HRV-induced lung inflammation in mice can be increased by the overexpression of human GDF15 protein, which results in heightened viral replication and release in airway epithelial cells [111]. Collectively, these results demonstrate that GDF15 promotes lung inflammation after cigarette smoke exposure. In addition, persistent active epithelial–mesenchymal transition (EMT) has been observed in the airway epithelial cells of patients with COPD; specifically, IL-17A markedly up-regulated the expression of GDF15 in cigarette smoke-treated HSAEpiC cells in a dose-and time-dependent manner, and IL-17A combined with GDF15 activated EMT in HSAEpiC cells after cigarette smoke exposure [112].

#### Pulmonary hypertension

In animal models of hypoxia, GDF15 overexpression in pulmonary vascular endothelial cells in pulmonary arterial hypertension (PAH) is accompanied by an elevated circulating GDF15 level, reflecting the process of pulmonary vascular remodelling [113, 114]. GDF15 promotes HIF-1α activation through p53 degradation, followed by the induction of angiogenesis in hypoxia-induced human umbilical vein endothelial cells (HUVECs) [115]. GDF15 could also prevent high glucose-induced endothelial cell apoptosis in HUVECs by inhibiting the phosphorylation of the PI3K/Akt/eNOS pathway and attenuating the activation of the NF-κB/JNK pathway [116]. An elevated systemic GDF15 level is associated with the risk, progression, and severity of pulmonary hypertension by increasing atrial and pulmonary capillary wedge pressure, which are caused by hypoxia and laminar shear stress in pulmonary vascular endothelial cells [114, 117]. GDF15 is also associated with left ventricular dysfunction as induced by pulmonary hypertension, especially in the case of persistent heart disease. As left heart disease leads to an increase in cell death and remodelling at the myocardial level, elevated GDF15 level can be used as a marker for the evaluation of post-capillary pulmonary hypertension [117]. In a study of children with congenital heart disease complicated by pulmonary hypertension, the GDF15 serum level was found to be considerably increased, which was positively associated with the level of N-terminal pro-brain natriuretic peptide (NT-proBNP). Furthermore, the addition of GDF15 to NT-proBNP as a diagnostic marker showed slightly higher specificity and positive predictive value than the use of NT-proBNP alone when diagnosing PAH [118]. In 2019, Larissi et al. [119] reported that patients with sickle cell disease had a high level of serum GDF15, with clinical manifestations of vascular occlusion, chronic haemolytic anaemia, and frequent infection, and the GDF15 level in the serum positively correlated with the mean pulmonary artery pressure. Tantawy et al. confirmed through echocardiography that young patients with thalassemia intermedia may have endothelial dysfunction present before the appearance of obvious clinical cardiovascular abnormalities, and this was accompanied by an increase in circulating GDF15 levels. They suggested using 1500 pg/mL GDF15 as a baseline to assess the presence of cardiovascular disease [120]. As a severe complication of systemic sclerosis, PAH is characterized by a high incidence and mortality rate, and GDF15 is significantly elevated in the remodelled pulmonary arteries and serum of systemic sclerosis-PAH patients [114].

In recent years, PAH has been increasingly regarded as a systemic disease. GDF15 has been linked to muscle atrophy in malignancy and anorexia nervosa. GDF15 inhibits appetite through its central receptor GFRAL, resulting in weight and muscle mass loss, and accelerates muscle protein degradation by up-regulating the expression of ubiquitin ligase atrogin-1, TAK1-NF-κB, and MuRF1 [113, 121]. The main muscle-related complications in PAH are declines in muscle strength, endurance, contractility, and capillary density along with the impaired oxygenation of microcirculation and a transition to type 2 muscle fibres [122]. GDF15 has been shown to contribute to muscle atrophy by increasing the phosphorylation of TAK1 and its target protein, NF-κB, and this process could be antagonised by treatment with TAK1 inhibitors [113]. This finding not only indicates that GDF15 is implicated in the pathogenesis of PAH vascular lesions but also shows that the pulmonary circulation affects the muscle mass of patients with PAH through a GDF15-mediated endocrine mechanism. In conclusion, GDF15 may participate in the pathogenesis of PAH vascular lesions and may be a powerful and promising biomarker for disease risk, progression, and a poor prognosis.

#### GDF15 and other lung diseases

GDF15 is also involved in the aetiology of other lung diseases. It has been found to promote and maintain T helper cell 2 immunity in the lung. In an asthma model mediated by allergens and environmental pollutant particles, NOTCH4 signalling up-regulated the expression of GDF15 in regulatory T cells, which promoted ILC2 expansion and activation through the Notch4-Wnt-GDF15 pathway [123] and provided a new therapeutic prospect for restoring lung immune tolerance and homeostasis [124].

In a retrospective cohort study of patients with acute respiratory distress syndrome, a higher level of GDF15 was strongly associated with a poor prognosis [125]. Herter et al. [126] found that GDF15 could protect the lungs of patients with acute lung injury by reducing the platelet count and suppressing neutrophil extracellular trap formation via the activation of αIIBβ3 on platelets. GDF15 also improved lung injury by up-regulating SIRT1 in an LPS-induced acute lung injury mouse model [127]. In addition, GDF15 has been found to affect the recruitment of neutrophils in the post-capillary venules of the cremaster muscle in a ventilator-induced lung injury model.

Dynamic changes in the GDF15 level are furthermore closely associated with COVID-19 progression, and they are used as a useful marker for identifying patients with poor respiratory function [128, 129]. Therefore, GDF15 may be used as an index to evaluate disease severity in patients with COVID-19. The pathogenesis of severe COVID-19 involves an overactive immune response, leading to a ‘cytokine storm’ characterised by haemophagocytosis and elevated serum cytokine levels [130]. Moreover, as SARS-CoV-2 directly targets endothelial cells, endothelial dysfunction is a trait of COVID-19 that is related to oxidative stress [129]. Considering its capacity to induce hypoxia and characteristic high expression in endothelial cells, GDF15 may also participate in COVID-19 endothelial inflammation [114, 129, 131]. Another possible mechanism underlying the oxidative stress and inflammation in COVID-19 involves a disorder of iron metabolism [132]. GDF15 has been reported as an upstream negative regulator of hepcidin, being associated with hepcidin levels and/or regulation of hepcidin expression. The plasma GDF15 level is higher in thalassemia and other diseases with ineffective erythropoiesis [119, 133]. The negative correlation between GDF15 and hepcidin [134], which may be associated with the Smad signal pathway, results in iron overload [135]. In addition, inhibition of GDF15 can also promote erastin-induced ferroptosis by attenuating the expression of SLC7A11. Therefore, GDF15 may play a key role in regulating ferroptosis and iron metabolism [136].

## Conclusions

In cells, GDF15 is present in different forms, with mature GDF15 being distributed in various human organs. As a molecule closely associated with stress and the ageing process, GDF15 is linked to the pathogenesis of several lung diseases, particularly chronic lung diseases; however, several inconsistencies remain with the molecular mechanism underlying GDF15 function at the cellular level. Although other lung cells may also secrete GDF15 under different disease or stress conditions, pulmonary epithelial cells, which are the most likely source of GDF15 in the lungs, play a role in subsequent immune responses, such as oxidative stress and inflammation. GDF15 is considered a core SASP protein and mitokine that is strongly associated with mitochondrial dysfunction. Furthermore, GDF15 has been identified as a potential biomarker for assessing the degree of mitochondrial dysfunction in ageing and age-related diseases. As a secreted protein, GDF15 can be used as not only a predictor of all-cause mortality but also a biomarker for the diagnosis, progression assessment, and prognosis of various lung diseases. Future research may be required on the pathogenesis of GDF15 in chronic lung diseases, such as the identification of (i) the upstream molecules involved in regulating GDF15 expression, (ii) receptors in lung tissues that directly bind GDF15, and (iii) mechanisms underlying the association between GDF15 and ageing, as well as mitochondrial dysfunction in the development of chronic lung diseases.

## Data Availability

Data sharing is not applicable to this article as no datasets were generated or analysed during the current study.

## References

[1] Bootcov MR, Bauskin AR, Valenzuela SM, Moore AG, Bansal M, He XY, Zhang HP, Donnellan M, Mahler S, Pryor K, Walsh BJ, Nicholson RC, Fairlie WD, Por SB, Robbins JM, Breit SN (1997). MIC-1, a novel macrophage inhibitory cytokine, is a divergent member of the TGF-beta superfamily. Proc Natl Acad Sci USA.

[2] Hromas R, Hufford M, Sutton J, Xu D, Li Y, Lu L (1997). PLAB, a novel placental bone morphogenetic protein. Biochim Biophys Acta.

[3] Lawton LN, Bonaldo MF, Jelenc PC, Qiu L, Baumes SA, Marcelino RA, de Jesus GM, Wellington S, Knowles JA, Warburton D, Brown S, Soares MB (1997). Identification of a novel member of the TGF-beta superfamily highly expressed in human placenta. Gene.

[4] Assadi A, Zahabi A, Hart RA (2020). GDF15, an update of the physiological and pathological roles it plays: a review. Pflugers Arch.

[5] Böttner M, Laaff M, Schechinger B, Rappold G, Unsicker K, Suter-Crazzolara C (1999). Characterization of the rat, mouse, and human genes of growth/differentiation factor-15/macrophage inhibiting cytokine-1 (GDF-15/MIC-1). Gene.

[6] Hsu JY, Crawley S, Chen M, Ayupova DA, Lindhout DA, Higbee J, Kutach A, Joo W, Gao Z, Fu D, To C, Mondal K, Li B, Kekatpure A, Wang M, Laird T, Horner G, Chan J, McEntee M, Lopez M, Lakshminarasimhan D, White A, Wang SP, Yao J, Yie J, Matern H, Solloway M, Haldankar R, Parsons T, Tang J, Shen WD, Chen YA, Tian H, Allan BB (2017). Non-homeostatic body weight regulation through a brainstem-restricted receptor for GDF15. Nature.

[7] Wang X, Baek SJ, Eling TE (2013). The diverse roles of nonsteroidal anti-inflammatory drug activated gene (NAG-1/GDF15) in cancer. Biochem Pharmacol.

[8] Fairlie WD, Zhang HP, Wu WM, Pankhurst SL, Bauskin AR, Russell PK, Brown PK, Breit SN (2001). The propeptide of the transforming growth factor-beta superfamily member, macrophage inhibitory cytokine-1 (MIC-1), is a multifunctional domain that can facilitate protein folding and secretion. J Biol Chem.

[9] Baek SJ, Eling T (2019). Growth differentiation factor 15 (GDF15): a survival protein with therapeutic potential in metabolic diseases. Pharmacol Ther.

[10] Kempf T, Horn-Wichmann R, Brabant G, Peter T, Allhoff T, Klein G, Drexler H, Johnston N, Wallentin L, Wollert KC (2007). Circulating concentrations of growth-differentiation factor 15 in apparently healthy elderly individuals and patients with chronic heart failure as assessed by a new immunoradiometric sandwich assay. Clin Chem.

[11] Moore AG, Brown DA, Fairlie WD, Bauskin AR, Brown PK, Munier ML, Russell PK, Salamonsen LA, Wallace EM, Breit SN (2000). The transforming growth factor-ss superfamily cytokine macrophage inhibitory cytokine-1 is present in high concentrations in the serum of pregnant women. J Clin Endocrinol Metab.

[12] Luan HH, Wang A, Hilliard BK, Carvalho F, Rosen CE, Ahasic AM, Herzog EL, Kang I, Pisani MA, Yu S, Zhang C, Ring AM, Young LH, Medzhitov R (2019). GDF15 is an inflammation-induced central mediator of tissue tolerance. Cell.

[13] Han ES, Muller FL, Pérez VI, Qi W, Liang H, Xi L, Fu C, Doyle E, Hickey M, Cornell J, Epstein CJ, Roberts LJ, Van Remmen H, Richardson A (2008). The in vivo gene expression signature of oxidative stress. Physiol Genom.

[14] Zheng H, Wu Y, Guo T, Liu F, Xu Y, Cai S (2020). Hypoxia induces growth differentiation factor 15 to promote the metastasis of colorectal cancer via PERK-eIF2α signaling. Biomed Res Int.

[15] Albertoni M, Shaw PH, Nozaki M, Godard S, Tenan M, Hamou MF, Fairlie DW, Breit SN, Paralkar VM, de Tribolet N, Van Meir EG, Hegi ME (2002). Anoxia induces macrophage inhibitory cytokine-1 (MIC-1) in glioblastoma cells independently of p53 and HIF-1. Oncogene.

[16] Al-Mudares F, Reddick S, Ren J, Venkatesh A, Zhao C, Lingappan K (2020). Role of growth differentiation factor 15 in lung disease and senescence: potential role across the lifespan. Front Med.

[17] Wang D, Day EA, Townsend LK, Djordjevic D, Jørgensen SB, Steinberg GR (2021). GDF15: emerging biology and therapeutic applications for obesity and cardiometabolic disease. Nat Rev Endocrinol.

[18] Krieg AJ, Rankin EB, Chan D, Razorenova O, Fernandez S, Giaccia AJ (2010). Regulation of the histone demethylase JMJD1A by hypoxia-inducible factor 1 alpha enhances hypoxic gene expression and tumor growth. Mol Cell Biol.

[19] Baek SJ, Kim JS, Moore SM, Lee SH, Martinez J, Eling TE (2005). Cyclooxygenase inhibitors induce the expression of the tumor suppressor gene EGR-1, which results in the up-regulation of NAG-1, an antitumorigenic protein. Mol Pharmacol.

[20] Baek SJ, Eling TE (2006). Changes in gene expression contribute to cancer prevention by COX inhibitors. Prog Lipid Res.

[21] Yang H, Park SH, Choi HJ, Moon Y (2010). The integrated stress response-associated signals modulates intestinal tumor cell growth by NSAID-activated gene 1 (NAG-1/MIC-1/PTGF-beta). Carcinogenesis.

[22] Baek SJ, Wilson LC, Hsi LC, Eling TE (2003). Troglitazone, a peroxisome proliferator-activated receptor gamma (PPAR gamma) ligand, selectively induces the early growth response-1 gene independently of PPAR gamma. A novel mechanism for its anti-tumorigenic activity. J Biol Chem.

[23] Yang H, Choi HJ, Park SH, Kim JS, Moon Y (2009). Macrophage inhibitory cytokine-1 (MIC-1) and subsequent urokinase-type plasminogen activator mediate cell death responses by ribotoxic anisomycin in HCT-116 colon cancer cells. Biochem Pharmacol.

[24] Li A, Zhao F, Zhao Y, Liu H, Wang Z (2021). ATF4-mediated GDF15 suppresses LPS-induced inflammation and MUC5AC in human nasal epithelial cells through the PI3K/Akt pathway. Life Sci.

[25] Kim Y, Noren Hooten N, Evans MK (2018). CRP stimulates GDF15 expression in endothelial cells through p53. Mediators Inflamm.

[26] Ratnam NM, Peterson JM, Talbert EE, Ladner KJ, Rajasekera PV, Schmidt CR, Dillhoff ME, Swanson BJ, Haverick E, Kladney RD, Williams TM, Leone GW, Wang DJ, Guttridge DC (2017). NF-κB regulates GDF-15 to suppress macrophage surveillance during early tumor development. J Clin Invest.

[27] Johnen H, Lin S, Kuffner T, Brown DA, Tsai VW, Bauskin AR, Wu L, Pankhurst G, Jiang L, Junankar S, Hunter M, Fairlie WD, Lee NJ, Enriquez RF, Baldock PA, Corey E, Apple FS, Murakami MM, Lin EJ, Wang C, During MJ, Sainsbury A, Herzog H, Breit SN (2007). Tumor-induced anorexia and weight loss are mediated by the TGF-beta superfamily cytokine MIC-1. Nat Med.

[28] Mullican SE, Lin-Schmidt X, Chin CN, Chavez JA, Furman JL, Armstrong AA, Beck SC, South VJ, Dinh TQ, Cash-Mason TD, Cavanaugh CR, Nelson S, Huang C, Hunter MJ, Rangwala SM (2017). GFRAL is the receptor for GDF15 and the ligand promotes weight loss in mice and nonhuman primates. Nat Med.

[29] Yang L, Chang CC, Sun Z, Madsen D, Zhu H, Padkjær SB, Wu X, Huang T, Hultman K, Paulsen SJ, Wang J, Bugge A, Frantzen JB, Nørgaard P, Jeppesen JF, Yang Z, Secher A, Chen H, Li X, John LM, Shan B, He Z, Gao X, Su J, Hansen KT, Yang W, Jørgensen SB (2017). GFRAL is the receptor for GDF15 and is required for the anti-obesity effects of the ligand. Nat Med.

[30] Emmerson PJ, Wang F, Du Y, Liu Q, Pickard RT, Gonciarz MD, Coskun T, Hamang MJ, Sindelar DK, Ballman KK, Foltz LA, Muppidi A, Alsina-Fernandez J, Barnard GC, Tang JX, Liu X, Mao X, Siegel R, Sloan JH, Mitchell PJ, Zhang BB, Gimeno RE, Shan B, Wu X (2017). The metabolic effects of GDF15 are mediated by the orphan receptor GFRAL. Nat Med.

[31] Breit SN, Brown DA, Tsai VW (2021). The GDF15-GFRAL pathway in health and metabolic disease: friend or foe?. Annu Rev Physiol.

[32] Takahashi M (2022). RET receptor signaling: function in development, metabolic disease, and cancer. Proc Jpn Acad Ser B Phys Biol Sci.

[33] Suriben R, Chen M, Higbee J, Oeffinger J, Ventura R, Li B, Mondal K, Gao Z, Ayupova D, Taskar P, Li D, Starck SR, Chen HIH, McEntee M, Katewa SD, Phung V, Wang M, Kekatpure A, Lakshminarasimhan D, White A, Olland A, Haldankar R, Solloway MJ, Hsu JY, Wang Y, Tang J, Lindhout DA, Allan BB (2020). Antibody-mediated inhibition of GDF15-GFRAL activity reverses cancer cachexia in mice. Nat Med.

[34] Zhang Y, Zhang G, Liu Y, Chen R, Zhao D, McAlister V, Mele T, Liu K, Zheng X (2018). GDF15 regulates Malat-1 circular RNA and inactivates NFκB signaling leading to immune tolerogenic DCs for preventing alloimmune rejection in heart transplantation. Front Immunol.

[35] Zhang Y, Jiang M, Nouraie M, Roth MG, Tabib T, Winters S, Chen X, Sembrat J, Chu Y, Cardenes N, Tuder RM, Herzog EL, Ryu C, Rojas M, Lafyatis R, Gibson KF, McDyer JF, Kass DJ, Alder JK (2019). GDF15 is an epithelial-derived biomarker of idiopathic pulmonary fibrosis. Am J Physiol Lung Cell Mol Physiol.

[36] Wu Q, Jiang D, Matsuda JL, Ternyak K, Zhang B, Chu HW (2016). Cigarette smoke induces human airway epithelial senescence via growth differentiation factor 15 production. Am J Respir Cell Mol Biol.

[37] Xu J, Kimball TR, Lorenz JN, Brown DA, Bauskin AR, Klevitsky R, Hewett TE, Breit SN, Molkentin JD (2006). GDF15/MIC-1 functions as a protective and antihypertrophic factor released from the myocardium in association with SMAD protein activation. Circ Res.

[38] Heger J, Schiegnitz E, von Waldthausen D, Anwar MM, Piper HM, Euler G (2010). Growth differentiation factor 15 acts anti-apoptotic and pro-hypertrophic in adult cardiomyocytes. J Cell Physiol.

[39] Li S, Ma YM, Zheng PS, Zhang P (2018). GDF15 promotes the proliferation of cervical cancer cells by phosphorylating AKT1 and ERK1/2 through the receptor ErbB2. J Exp Clin Cancer Res.

[40] Adela R, Banerjee SK (2015). GDF-15 as a target and biomarker for diabetes and cardiovascular diseases: a translational prospective. J Diabetes Res.

[41] Hsiao EC, Koniaris LG, Zimmers-Koniaris T, Sebald SM, Huynh TV, Lee SJ (2000). Characterization of growth-differentiation factor 15, a transforming growth factor beta superfamily member induced following liver injury. Mol Cell Biol.

[42] Zimmers TA, Jin X, Hsiao EC, McGrath SA, Esquela AF, Koniaris LG (2005). Growth differentiation factor-15/macrophage inhibitory cytokine-1 induction after kidney and lung injury. Shock (Augusta, GA).

[43] Kempf T, Eden M, Strelau J, Naguib M, Willenbockel C, Tongers J, Heineke J, Kotlarz D, Xu J, Molkentin JD, Niessen HW, Drexler H, Wollert KC (2006). The transforming growth factor-beta superfamily member growth-differentiation factor-15 protects the heart from ischemia/reperfusion injury. Circ Res.

[44] Xu XY, Nie Y, Wang FF, Bai Y, Lv ZZ, Zhang YY, Li ZJ, Gao W (2014). Growth differentiation factor (GDF)-15 blocks norepinephrine-induced myocardial hypertrophy via a novel pathway involving inhibition of epidermal growth factor receptor transactivation. J Biol Chem.

[45] Lambert JR, Whitson RJ, Iczkowski KA, La Rosa FG, Smith ML, Wilson RS, Smith EE, Torkko KC, Gari HH, Lucia MS (2015). Reduced expression of GDF-15 is associated with atrophic inflammatory lesions of the prostate. Prostate.

[46] Abulizi P, Loganathan N, Zhao D, Mele T, Zhang Y, Zwiep T, Liu K, Zheng X (2017). Growth differentiation Factor-15 deficiency augments inflammatory response and exacerbates septic heart and renal injury induced by lipopolysaccharide. Sci Rep.

[47] Li M, Song K, Huang X, Fu S, Zeng Q (2018). GDF 15 prevents LPS and D galactosamine induced inflammation and acute liver injury in mice. Int J Mol Med.

[48] Santos I, Colaço HG, Neves-Costa A, Seixas E, Velho TR, Pedroso D, Barros A, Martins R, Carvalho N, Payen D, Weis S, Yi HS, Shong M, Moita LF (2020). CXCL5-mediated recruitment of neutrophils into the peritoneal cavity of Gdf15-deficient mice protects against abdominal sepsis. Proc Natl Acad Sci USA.

[49] Wiklund FE, Bennet AM, Magnusson PK, Eriksson UK, Lindmark F, Wu L, Yaghoutyfam N, Marquis CP, Stattin P, Pedersen NL, Adami HO, Grönberg H, Breit SN, Brown DA (2010). Macrophage inhibitory cytokine-1 (MIC-1/GDF15): a new marker of all-cause mortality. Aging Cell.

[50] Pence BD, Yarbro JR, Emmons RS (2020). Growth differentiation factor-15 is associated with age-related monocyte dysfunction. Aging Med (Milton).

[51] Basisty N, Kale A, Jeon OH, Kuehnemann C, Payne T, Rao C, Holtz A, Shah S, Sharma V, Ferrucci L, Campisi J, Schilling B (2020). A proteomic atlas of senescence-associated secretomes for aging biomarker development. PLOS Biol.

[52] Park H, Kim CH, Jeong JH, Park M, Kim KS (2016). GDF15 contributes to radiation-induced senescence through the ROS-mediated p16 pathway in human endothelial cells. Oncotarget.

[53] Wang X, Chrysovergis K, Kosak J, Kissling G, Streicker M, Moser G, Li R, Eling TE (2014). hNAG-1 increases lifespan by regulating energy metabolism and insulin/IGF-1/mTOR signaling. Aging.

[54] Yatsuga S, Fujita Y, Ishii A, Fukumoto Y, Arahata H, Kakuma T, Kojima T, Ito M, Tanaka M, Saiki R, Koga Y (2015). Growth differentiation factor 15 as a useful biomarker for mitochondrial disorders. Ann Neurol.

[55] Morrow RM, Picard M, Derbeneva O, Leipzig J, McManus MJ, Gouspillou G, Barbat-Artigas S, Dos Santos C, Hepple RT, Murdock DG, Wallace DC (2017). Mitochondrial energy deficiency leads to hyperproliferation of skeletal muscle mitochondria and enhanced insulin sensitivity. Proc Natl Acad Sci USA.

[56] Melber A, Haynes CM (2018). UPRmt regulation and output: a stress response mediated by mitochondrial-nuclear communication. Cell Res.

[57] Kim KH, Lee MS (2021). GDF15 as a central mediator for integrated stress response and a promising therapeutic molecule for metabolic disorders and NASH. Biochim Biophys Acta Gen Subj.

[58] Quirós PM, Prado MA, Zamboni N, D’Amico D, Williams RW, Finley D, Gygi SP, Auwerx J (2017). Multi-omics analysis identifies ATF4 as a key regulator of the mitochondrial stress response in mammals. J Cell Biol.

[59] Fiorese CJ, Schulz AM, Lin YF, Rosin N, Pellegrino MW, Haynes CM (2016). The transcription factor ATF5 mediates a mammalian mitochondrial UPR. Curr Biol.

[60] Michel S, Canonne M, Arnould T, Renard P (2015). Inhibition of mitochondrial genome expression triggers the activation of CHOP-10 by a cell signaling dependent on the integrated stress response but not the mitochondrial unfolded protein response. Mitochondrion.

[61] Rochette L, Meloux A, Zeller M, Malka G, Cottin Y, Vergely C (2020). Mitochondrial SLC25 carriers: novel targets for cancer therapy. Molecules.

[62] Chung HK, Ryu D, Kim KS, Chang JY, Kim YK, Yi HS, Kang SG, Choi MJ, Lee SE, Jung SB, Ryu MJ, Kim SJ, Kweon GR, Kim H, Hwang JH, Lee CH, Lee SJ, Wall CE, Downes M, Evans RM, Auwerx J, Shong M (2017). Growth differentiation factor 15 is a myomitokine governing systemic energy homeostasis. J Cell Biol.

[63] Kang SG, Choi MJ, Jung SB, Chung HK, Chang JY, Kim JT, Kang YE, Lee JH, Hong HJ, Jun SM, Ro HJ, Suh JM, Kim H, Auwerx J, Yi HS, Shong M (2021). Differential roles of GDF15 and FGF21 in systemic metabolic adaptation to the mitochondrial integrated stress response. Science.

[64] Townsend LK, Weber AJ, Day EA, Shamshoum H, Shaw SJ, Perry CGR, Kemp BE, Steinberg GR, Wright DC (2021). AMPK mediates energetic stress-induced liver GDF15. FASEB J.

[65] Klaus S, Ost M (2020). Mitochondrial uncoupling and longevity - A role for mitokines?. Exp Gerontol.

[66] Liu H, Liu J, Si L, Guo C, Liu W, Liu Y (2019). GDF-15 promotes mitochondrial function and proliferation in neuronal HT22 cells. J Cell Biochem.

[67] Wang Y, Chen C, Chen J, Sang T, Peng H, Lin X, Zhao Q, Chen S, Eling T, Wang X (2022). Overexpression of NAG-1/GDF15 prevents hepatic steatosis through inhibiting oxidative stress-mediated dsDNA release and AIM2 inflammasome activation. Redox Biol.

[68] Li P, Lv H, Zhang B, Duan R, Zhang X, Lin P, Song C, Liu Y (2022). Growth Differentiation factor 15 protects SH-SY5Y cells from rotenone-induced toxicity by suppressing mitochondrial apoptosis. Front Aging Neurosci.

[69] Hansmann G, Sallmon H, Roehr CC, Kourembanas S, Austin ED, Koestenberger M, European Pediatric Pulmonary Vascular Disease Network (EPPVDN) (2021). Pulmonary hypertension in bronchopulmonary dysplasia. Pediatr Res.

[70] Jensen EA, Dysart K, Gantz MG, McDonald S, Bamat NA, Keszler M, Kirpalani H, Laughon MM, Poindexter BB, Duncan AF, Yoder BA, Eichenwald EC, DeMauro SB (2019). The diagnosis of bronchopulmonary dysplasia in very preterm infants. An evidence-based approach. Am J Respir Crit Care Med.

[71] Meiners S, Hilgendorff A (2016). Early injury of the neonatal lung contributes to premature lung aging: a hypothesis. Mol Cell Pediatr.

[72] Meiners S, Eickelberg O, Königshoff M (2015). Hallmarks of the ageing lung. Eur Respir J.

[73] Hilgendorff A, O’Reilly MA (2015). Bronchopulmonary dysplasia early changes leading to long-term consequences. Front Med (Lausanne).

[74] Kunzmann S, Speer CP, Jobe AH, Kramer BW (2007). Antenatal inflammation induced TGF-beta1 but suppressed CTGF in preterm lungs. Am J Physiol Lung Cell Mol Physiol.

[75] Londhe VA, Sundar IK, Lopez B, Maisonet TM, Yu Y, Aghai ZH, Rahmanet I (2011). Hyperoxia impairs alveolar formation and induces senescence through decreased histone deacetylase activity and up-regulation of p21 in neonatal mouse lung. Pediatr Res.

[76] Sasaki T, Tahara S, Shinkai T, Kuramoto K, Matsumoto S, Yanabe M, Takagi S, Kondo H, Kaneko T (2013). Lifespan extension in the spontaneous dwarf rat and enhanced resistance to hyperoxia-induced mortality. Exp Gerontol.

[77] Volonte D, Galbiati F (2009). Caveolin-1, cellular senescence and pulmonary emphysema. Aging.

[78] Scaffa AM, Peterson AL, Carr JF, Garcia D, Yao H, Dennery PA (2021). Hyperoxia causes senescence and increases glycolysis in cultured lung epithelial cells. Physiol Rep.

[79] Parikh P, Britt RD, Manlove LJ, Wicher SA, Roesler A, Ravix J, Teske J, Thompson MA, Sieck GC, Kirkland JL, LeBrasseur N, Tschumperlin DJ, Pabelick CM, Prakash YS (2019). Hyperoxia-induced cellular senescence in fetal airway smooth muscle cells. Am J Respir Cell Mol Biol.

[80] Parikh P, Wicher S, Khandalavala K, Pabelick CM, Britt RD, Prakash YS (2019). Cellular senescence in the lung across the age spectrum. Am J Physiol Lung Cell Mol Physiol.

[81] Saretzki G, Feng J, von Zglinicki T, Villeponteau B (1998). Similar gene expression pattern in senescent and hyperoxic-treated fibroblasts. J Gerontol A Biol Sci Med Sci.

[82] Bhattacharya S, Zhou Z, Yee M, Chu CY, Lopez AM, Lunger VA, Solleti SK, Resseguie E, Buczynski B, Mariani TJ, O'Reilly MA (2014). The genome-wide transcriptional response to neonatal hyperoxia identifies Ahr as a key regulator. Am J Physiol Lung Cell Mol Physiol.

[83] Tiwari KK, Moorthy B, Lingappan K (2015). Role of GDF15 (growth and differentiation factor 15) in pulmonary oxygen toxicity. Toxicol In Vitro.

[84] Zhang Y, Jiang W, Wang L, Lingappan K (2017). Sex-specific differences in the modulation of growth differentiation Factor 15 (GDF15) by hyperoxia in vivo and in vitro: role of HIF-1alpha. Toxicol Appl Pharmacol.

[85] Díaz M, Campderrós L, Guimaraes MP, López-Bermejo A, de Zegher F, Villarroya F, Ibáñez L (2020). Circulating growth-and-differentiation factor-15 in early life: relation to prenatal and postnatal growth and adiposity measurements. Pediatr Res.

[86] Xiong Y, Walker K, Min X, Hale C, Tran T, Komorowski R, Yang J, Davda J, Nuanmanee N, Kemp D, Wang X, Liu H, Miller S, Lee KJ, Wang Z, Véniant MM (2017). Long-acting MIC-1/GDF15 molecules to treat obesity: evidence from mice to monkeys. Sci Transl Med.

[87] Almudares F, Hagan J, Chen X, Devaraj S, Moorthy B, Lingappan K (2023). Growth and differentiation factor 15 (GDF15) levels predict adverse respiratory outcomes in premature neonates. Pediatr Pulmonol.

[88] Takenouchi Y, Kitakaze K, Tsuboi K, Okamoto Y (2020). Growth differentiation factor 15 facilitates lung fibrosis by activating macrophages and fibroblasts. Exp Cell Res.

[89] Tian Y, Li H, Qiu T, Dai J, Zhang Y, Chen J, Cai H (2019). Loss of PTEN induces lung fibrosis via alveolar epithelial cell senescence depending on NF-κB activation. Aging Cell.

[90] Cao M, Gu L, Guo L, Liu M, Wang T, Zhang J, Zhang H, Zhang Y, Shi Y, Zhao Y, Qiu X, Gui X, Ma M, Tian Y, Liu X, Meng F, Xiao Y, Sun L (2022). Elevated expression of growth differentiation factor-15 is associated with acute exacerbation of idiopathic pulmonary fibrosis. Front Immunol.

[91] Radwanska A, Cottage CT, Piras A, Overed-Sayer C, Sihlbom C, Budida R, Wrench C, Connor J, Monkley S, Hazon P, Schluter H, Thomas MJ, Hogaboam CM, Murray LA (2022). Increased expression and accumulation of GDF15 in IPF extracellular matrix contribute to fibrosis. JCI Insight.

[92] Lambrecht S, Smith V, De Wilde K, Coudenys J, Decuman S, Deforce D, De Keyser F, Elewaut D (2014). Growth differentiation factor 15, a marker of lung involvement in systemic sclerosis, is involved in fibrosis development but is not indispensable for fibrosis development. Arthritis Rheumatol.

[93] Lee EK, Lee YS, Han IO, Park SH (2016). Expression of Caveolin-1 reduces cellular responses to TGF-beta1 through down-regulating the expression of TGF-beta type II receptor gene in NIH3T3 fibroblast cells. Biochem Biophys Res Commun.

[94] Budi EH, Xu J, Derynck R (2016). Regulation of TGF-β receptors. Methods Mol Biol.

[95] He K, Yan X, Li N, Dang S, Xu L, Zhao B, Li Z, Lv Z, Fang X, Zhang Y, Chen YG (2015). Internalization of the TGF-β type I receptor into caveolin-1 and EEA1 double-positive early endosomes. Cell Res.

[96] He J, Li X, Yu M (2021). Bioinformatics analysis identifies potential ferroptosis key genes in the pathogenesis of pulmonary fibrosis. Front Genet.

[97] Kim YI, Shin HW, Chun YS, Cho CH, Koh J, Chung DH, Park JW (2018). Epithelial cell-derived cytokines CST3 and GDF15 as potential therapeutics for pulmonary fibrosis. Cell Death Dis.

[98] Rabe KF, Watz H (2017). Chronic obstructive pulmonary disease. Lancet.

[99] Mueller T, Leitner I, Egger M, Haltmayer M, Dieplinger B (2015). Association of the Biomarkers soluble ST2, galectin-3 and growth-differentiation factor-15 with heart failure and other non-cardiac diseases. Clin Chim Acta.

[100] Mutlu LC, Altintas N, Aydin M, Tulubas F, Oran M, Kucukyalin V, Kaplan G, Gurel A (2015). Growth differentiation Factor-15 is a novel biomarker predicting acute exacerbation of chronic obstructive pulmonary disease. Inflammation.

[101] Kim M, Cha SI, Choi KJ, Shin KM, Lim JK, Yoo SS, Lee J, Lee SY, Kim CH, Park JY, Yang DH (2014). Prognostic value of serum growth differentiation factor-15 in patients with chronic obstructive pulmonary disease exacerbation. Tuberc Respir Dis.

[102] Hirano T, Doi K, Matsunaga K, Takahashi S, Donishi T, Suga K, Oishi K, Yasuda K, Mimura Y, Harada M, Suizu S, Murakawa K, Chikumoto A, Ohteru Y, Matsuda K, Uehara S, Hamada K, Ohata S, Murata Y, Yamaji Y, Asami-Noyama M, Edakuni N, Kakugawa T (2020). A novel role of growth differentiation factor (GDF)-15 in overlap with sedentary lifestyle and cognitive risk in COPD. J Clin Med.

[103] Husebø GR, Grønseth R, Lerner L, Gyuris J, Hardie JA, Bakke PS, Eagan TM (2017). Growth differentiation factor-15 is a predictor of important disease outcomes in patients with COPD. Eur Respir J.

[104] Martinez CH, Freeman CM, Nelson JD, Murray S, Wang X, Budoff MJ, Dransfield MT, Hokanson JE, Kazerooni EA, Kinney GL, Regan EA, Wells JM, Martinez FJ, Han MK, Curtis JL, COPD Gene Investigators (2017). GDF-15 plasma levels in chronic obstructive pulmonary disease are associated with subclinical coronary artery disease. Respir Res.

[105] Aoshiba K, Nagai A (2009). Senescence hypothesis for the pathogenetic mechanism of chronic obstructive pulmonary disease. Proc Am Thorac Soc.

[106] Zhou F, Onizawa S, Nagai A, Aoshiba K (2011). Epithelial cell senescence impairs repair process and exacerbates inflammation after airway injury. Respir Res.

[107] Yao H, Sundar IK, Gorbunova V, Rahman I (2013). P21-PARP-1 pathway is involved in cigarette smoke-induced lung DNA damage and cellular senescence. PLoS ONE.

[108] Tsuji T, Aoshiba K, Nagai A (2006). Alveolar cell senescence in patients with pulmonary emphysema. Am J Respir Crit Care Med.

[109] Wu Q, Jiang D, Chu HW (2012). Cigarette smoke induces growth differentiation factor 15 production in human lung epithelial cells: implication in mucin over-expression. Innate Immun.

[110] Verhamme FM, Seys LJM, De Smet EG, Provoost S, Janssens W, Elewaut D, Joos GF, Brusselle GG, Bracke KR (2017). Elevated GDF-15 contributes to pulmonary inflammation upon cigarette smoke exposure. Mucosal Immunol.

[111] Wu Q, Jiang D, Schaefer NR, Harmacek L, O'Connor BP, Eling TE, Eickelberg O, Chu HW (2018). Overproduction of growth differentiation factor 15 promotes human rhinovirus infection and virus-induced inflammation in the lung. Am J Physiol Lung Cell Mol Physiol.

[112] Jiang G, Liu CT, Zhang WD (2018). IL-17A and GDF15 are able to induce epithelial-mesenchymal transition of lung epithelial cells in response to cigarette smoke. Exp Ther Med.

[113] Garfield BE, Crosby A, Shao D, Yang P, Read C, Sawiak S, Moore S, Parfitt L, Harries C, Rice M, Paul R, Ormiston ML, Morrell NW, Polkey MI, Wort SJ, Kemp PR (2019). Growth/differentiation factor 15 causes TGFbeta-activated kinase 1-dependent muscle atrophy in pulmonary arterial hypertension. Thorax.

[114] Nickel N, Jonigk D, Kempf T, Bockmeyer CL, Maegel L, Rische J, Laenger F, Lehmann U, Sauer C, Greer M, Welte T, Hoeper MM, Golpon HA (2011). GDF-15 is abundantly expressed in plexiform lesions in patients with pulmonary arterial hypertension and affects proliferation and apoptosis of pulmonary endothelial cells. Respir Res.

[115] Song H, Yin D, Liu Z (2012). GDF-15 promotes angiogenesis through modulating p53/HIF-1alpha signaling pathway in hypoxic human umbilical vein endothelial cells. Mol Biol Rep.

[116] Li J, Yang L, Qin W, Zhang G, Yuan J, Wang F (2013). Adaptive induction of growth differentiation factor 15 attenuates endothelial cell apoptosis in response to high glucose stimulus. PLoS ONE.

[117] Mirna M, Rohm I, Jirak P, Wernly B, Bäz L, Paar V, Kretzschmar D, Hoppe UC, Schulze PC, Lichtenauer M, Jung C, Franz M (2020). Analysis of novel cardiovascular biomarkers in patients with pulmonary hypertension (PH). Heart Lung Circ.

[118] Li G, Li Y, Tan XQ, Jia P, Zhao J, Liu D, Wang T, Liu B (2017). Plasma growth differentiation Factor-15 is a potential biomarker for pediatric pulmonary arterial hypertension associated with congenital heart disease. Pediatr Cardiol.

[119] Larissi K, Politou M, Margeli A, Poziopoulos C, Flevari P, Terpos E, Papassotiriou I, Voskaridou E (2019). The growth differentiation Factor-15 (GDF-15) levels are increased in patients with compound heterozygous sickle cell and beta-thalassemia (HbS/βthal), correlate with markers of hemolysis, iron burden, coagulation, endothelial dysfunction and pulmonary hypertension. Blood Cells Mol Dis.

[120] Tantawy AA, Adly AA, Ismail EA, Youssef OI, Ali ME (2015). Growth differentiation factor-15 in children and adolescents with thalassemia intermedia: relation to subclinical atherosclerosis and pulmonary vasculopathy. Blood Cells Mol Dis.

[121] Bloch SA, Lee JY, Syburra T, Rosendahl U, Griffiths MJ, Kemp PR, Polkey MI (2015). Increased expression of GDF-15 may mediate ICU-acquired weakness by down-regulating muscle microRNAs. Thorax.

[122] Lai YC, Provencher S, Goncharova EA (2019). TAKling GDF-15 and skeletal muscle atrophy in pulmonary hypertension: are we there yet?. Thorax.

[123] Harb H, Stephen-Victor E, Crestani E, Benamar M, Massoud A, Cui Y, Charbonnier LM, Arbag S, Baris S, Cunnigham A, Leyva-Castillo JM, Geha RS, Mousavi AJ, Guennewig B, Schmitz-Abe K, Sioutas C, Phipatanakul W, Chatila TA (2020). A regulatory T cell Notch4-GDF15 axis licenses tissue inflammation in asthma. Nat Immunol.

[124] Hammad H, Lambrecht BN (2020). Wnt and Hippo pathways in regulatory T cells: a NOTCH above in asthma. Nat Immunol.

[125] Clark BJ, Bull TM, Benson AB, Stream AR, Macht M, Gaydos J, Meadows C, Burnham EL, Moss M, ARDS Network Investigators (2013). Growth differentiation factor-15 and prognosis in acute respiratory distress syndrome: a retrospective cohort study. Crit Care.

[126] Herter JM, Kraft F, Van Aken H, Meersch M, Zarbock A, Rossaint J (2015). GDF-15 prevents ventilator-induced lung injury by inhibiting the formation of platelet-neutrophil aggregates. Thromb Haemost.

[127] Song H, Chen Q, Xie S, Huang J, Kang G (2020). GDF-15 prevents lipopolysaccharide-mediated acute lung injury via upregulating SIRT1. Biochem Biophys Res Commun.

[128] Alserawan L, Peñacoba P, Orozco Echevarría SE, Castillo D, Ortiz E, Martínez-Martínez L, Naranjo EM, Domingo P, Castellví I, Juárez C, Mariscal A (2021). Growth differentiation Factor 15 (GDF-15): a novel biomarker associated with poorer respiratory function in COVID-19. Diagnostics.

[129] Ahmed DS, Isnard S, Berini C, Lin J, Routy JP, Royston L (2022). Coping with stress: the mitokine GDF-15 as a biomarker of COVID-19 severity. Front Immunol.

[130] Fajgenbaum DC, June CH (2020). Cytokine storm. N Engl J Med.

[131] Notz Q, Schmalzing M, Wedekink F, Schlesinger T, Gernert M, Herrmann J, Sorger L, Weismann D, Schmid B, Sitter M, Schlegel N, Kranke P, Wischhusen J, Meybohm P, Lotz C (2020). Pro- and anti-inflammatory responses in severe COVID-19-induced acute respiratory distress syndrome-an observational pilot study. Front Immunol.

[132] Rochette L, Zeller M, Cottin Y, Vergely C (2021). GDF15: an emerging modulator of immunity and a strategy in COVID-19 in association with iron metabolism. Trends Endocrinol Metab.

[133] Tanno T, Noel P, Miller JL (2010). Growth differentiation factor 15 in erythroid health and disease. Curr Opin Hematol.

[134] Rochette L, Gudjoncik A, Guenancia C, Zeller M, Cottin Y, Vergely C (2015). The iron-regulatory hormone hepcidin: a possible therapeutic target?. Pharmacol Ther.

[135] Tanno T, Porayette P, Sripichai O, Noh SJ, Byrnes C, Bhupatiraju A, Lee YT, Goodnough JB, Harandi O, Ganz T, Paulson RF, Miller JL (2009). Identification of TWSG1 as a second novel erythroid regulator of hepcidin expression in murine and human cells. Blood.

[136] Chen L, Qiao L, Bian Y, Sun X (2020). GDF15 knockdown promotes erastin-induced ferroptosis by decreasing SLC7A11 expression. Biochem Biophys Res Commun.

